# Addition of Lubiprostone to polyethylene glycol(PEG) enhances the quality & efficacy of colonoscopy preparation: a randomized, double-blind, placebo controlled trial

**DOI:** 10.1186/s12876-016-0542-0

**Published:** 2016-10-13

**Authors:** Rupa Banerjee, Hrushikesh Chaudhari, Nirish Shah, Arjunan Saravanan, Manu Tandan, D. Nageshwar Reddy

**Affiliations:** Department of Medical Gastroenterology, Asian Institute of Gastroenterology, 6-3-661, Somajiguda, Hyderabad, India

**Keywords:** Bowel preparation, Lubiprostone, Colonoscopy, PEG

## Abstract

**Background:**

Adequate bowel preparation is an essential prerequisite for complete mucosal visualization during colonoscopy. Polyethylene glycol (PEG) solutions are commonly used. However the large volume of the solution is often poorly tolerated. Addition of Lubiprostone (LB) could improve the adequacy of standard PEG preparation & reduce requirement. The aims to assess adequacy of PEG preparation with addition of single dose LB (24mcg) vs placebo and efficacy of reduced dose PEG + LB compared with full dose PEG + LB.

**Methods:**

Single center prospective double blind randomized controlled trial. Part I: 442 patients for colonoscopy randomized to receive placebo (GrA) or single dose of LB (GrB) prior to PEG preparation. Quality of bowel preparation graded 0–9 according to Boston Bowel Preparation Scale (BBPS). BBPS-9: excellent and BBPS 0–4: repeat procedure.

Part II: 146 patients randomized to receive LB + 1.5 L PEG (GrC; 75) or LB + 1 L PEG (GrD; 71). BBPS score compared with GrB (2 L PEG).

**Results:**

Part I: 442 patients (221 GrA & 221 Gr B). LB resulted in significant improvement in total BBPS (7.44 + 0.14 vs. 6.36 + 0.16, *p* < 0.0001). 66.5 % Gr B vs 38 % Gr A had excellent prep; 42.5 % GrB vs 24 % GrA had adequate prep. Repeat procedure needed 9.5 % Gr B vs 16.7 % Gr A (*P* < 0.01).

Part II: No difference in BBPS scores with lower doses (Gr C&D) compared to standard (GrB) (Mean BBPS 7.44 + 0.14 GrA,7.30 + 0.25 GrC;7.25 + 0.26 GrD;*p* >0.05).

**Conclusion:**

Single dose LB prior to PEG significantly enhanced bowel preparation compared to PEG alone. There was no significant difference in quality of preparation with lower doses of PEG when combined with LB.

**Trial registration:**

The study protocol was approved by institutional review board and the trial was registered on March 22, 2011 with clinicaltrials.gov (NCT01324284).

## Background

Colonoscopy is the current standard method for examination of the colon. Bowel cleansing prior to colonoscopy is the essential prerequisite to ensure complete mucosal visualization and lesion identification [1, 2]. Suboptimal preparations are associated with missed diagnoses, longer procedure times and increased costs related to the repeat procedures and shortened intervals between procedures [3–5]. In fact, poor bowel preparation and incomplete evaluation have accounted for a larger share of cancer risk than polyp characteristics for all defined population subgroups [6, 7]. Inadequate preparations have been noted in around 25 % cases in the US [4, 8]. This has been attributed primarily to poor patient tolerance to the standard colon preparations.

Osmotically balanced polyethylene glycol (PEG)- electrolyte bowel lavage solutions were introduced in 1980 [9]. These PEG based solutions are the most commonly used bowel preparations today [9]. They have high efficacy, are safe and are associated with minimal fluid and electrolyte imbalance. However the major drawback of these preparations is the taste and the large volumes required with associated nausea, cramping and vomiting [10]. This often results in poor compliance and tolerance with resultant poor preparation & improper visualization. The compliance rates for colonoscopy screening has been reported to be as low as 34 % in adults greater than 50 year of age [5]. A pooled analysis of 15 trials found that at least 29 % of patients were unable to complete their PEG solution [11].

Lubiprostone (LB) is a locally acting selective Type 2 chloride channel activator which causes intestinal fluid secretion. This results in increased softened stool and increased intestinal transit without the loss of either net intravascular fluid or electrolytes [12]. Peak plasma levels occur approximately 1.14 h after oral administration of a single 24 microgram dose, and the half-life of lubiprostone (t_½_) has been estimated at approximately 3 h [13, 14]. LB is currently approved for the treatment of chronic idiopathic constipation and is generally well tolerated with an excellent side effect profile. Even long term usage has not shown clinically significant changes in electrolyte levels [12, 15].

Our hypothesis was that administration of LB prior to bowel preparation would improve the adequacy of standard PEG preparation. Additionally it could reduce the dosage requirement of PEG and improve patient tolerability. Accordingly we conducted this prospective double blinded randomized placebo controlled trial. The primary objective was to evaluate the efficacy of standard PEG preparation with the addition of single dose LB (24mcg) compared to a placebo preparation. Subsequently we assessed the adequacy of reduced [1 l and 1.5 l (L)] doses of PEG preparation on addition of LB pretreatment.

## Methods

**Fig. 1:**
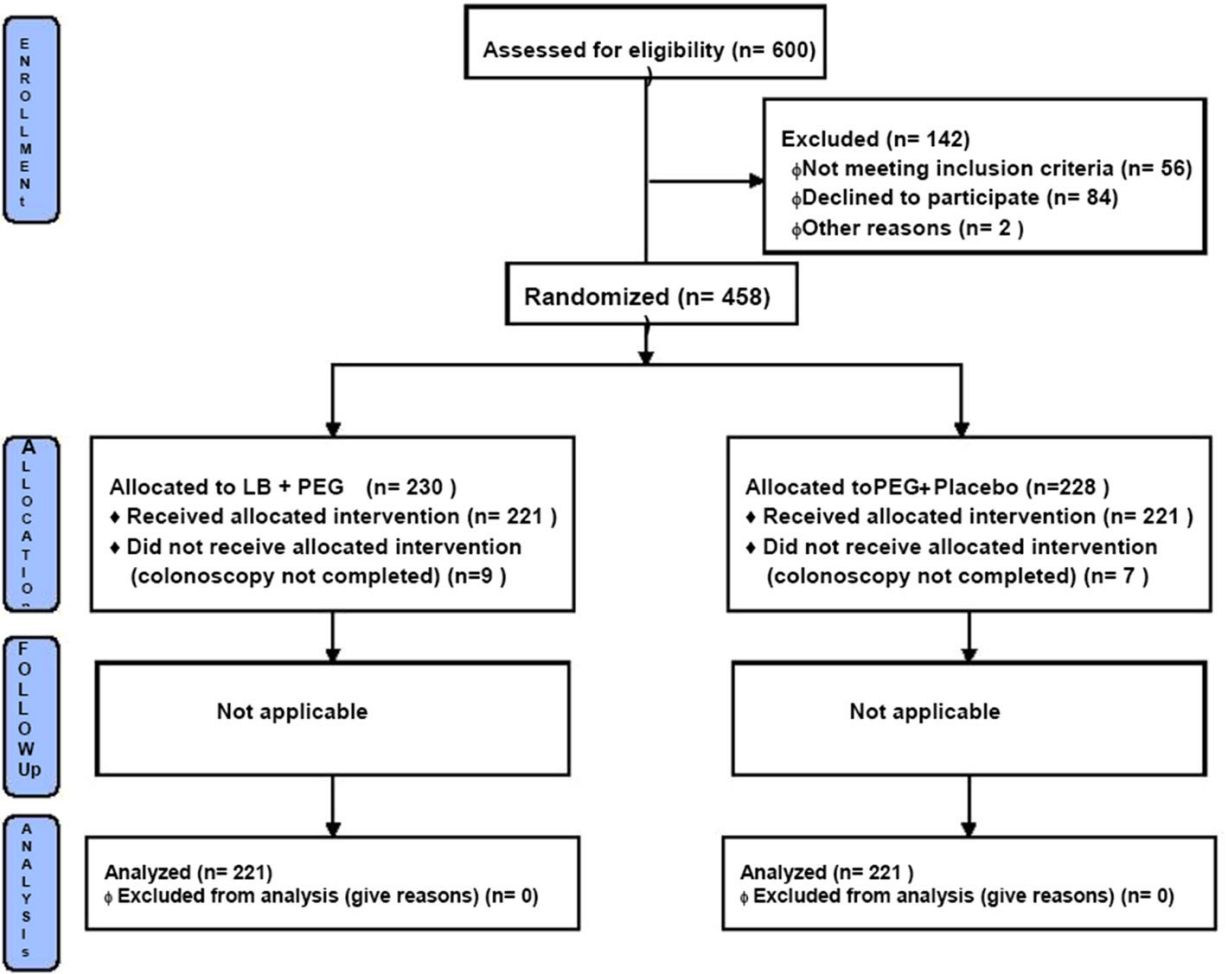
Consort flow diagram

This was a single center prospective double blind randomized controlled trial to compare quality of bowel preparation using PEG vs. PEG with LB. After observation of significant improvement in bowel preparation with addition of LB, we undertook second part of this study where we evaluated if addition of LB facilitated dose reduction of PEG. The study protocol was approved by institutional review board and the trial was registered with clinicaltrials.gov (NCT01324284). Recruitment, enrollment, randomization, withdrawal and completion were done according to the consort guidelines (Fig. 1).

### Participants

We prospectively offered enrollment to patients between ages of 18 and 75 years referred for outpatient colonoscopy to Asian Institute of Gastroenterology, Hyderabad from March to July 2011. Exclusion criteria included active GI bleeding, renal insufficiency (creatinine ≥ 1.6), dementia, recent myocardial infarction (in past 8 weeks), symptomatic heart failure (NYHA class C or D), gastroparesis, gastric outlet obstruction, ileus, toxic megacolon, suspected colonic obstruction, pregnancy and lactation. Informed consent was taken from the patients prior to enrolment.

### Randomization

All subjects attended an informational session before colonoscopy where they were counseled about the nature of study and an informed written consent was obtained. The Participants were randomized in two groups for the Part 1 study. The research coordinator used computer generated block randomization to allocate the patients in standard of care 2 L PEG group (Group A) or 2 L PEG with LB group(Group B) [16]. The patients in group A were given a placebo pill identical in size, shape and color to LB pill. In the second part of the study, the Participants were randomly assigned to 1.5 L PEG + LB (Group C) and 1 L PEG + LB (Group D). All patients, endoscopists and endoscopy nursing and support staff were blinded to patient allocation. All patients were given appropriate oral and written instructions regarding the lavage solution, tablets and diet.

### Bowel Preparation

#### Part 1

On the day of procedure, group B was given one 24 mcg tablet of LB (Sun Pharmaceutical Industries Ltd.). Group A was given a placebo identical in appearance to LB tablet which was also supplied by Sun pharmaceuticals Ltd. One hour after LB or placebo ingestion, the patients were given 2 L PEG solution with electrolytes (polyethylene glycol 118 g, sod. chloride 2.93 gm, potassium chloride 1.484 gm, sod bicarbonate 3.37 gm, anhydrous sodium sulfate 11.36 g) (Peglec, Tablets India Ltd). They were instructed to take 200 cc every 10 min till the entire solution was consumed. The subjects received standardized diet instructions which did not require any overnight fasting or special diet the day prior to the colonoscopy. They were instructed to take clear liquid diet while taking colonoscopy preparation followed by nil per oral (NPO) status till the end of procedure.

#### Part 2

After completion of Part 1 and analysis of results, two additional groups were added to investigate if decreasing PEG dose affects colonoscopy preparation. In the Part 2, all patients in group C and group D received 24 mcg of LB. Patients in group C were given 1.5 L of PEG solution while group D were given 1 L PEG one hour after taking LB. Standardized diet and colonoscopy lavage instructions were unchanged between Part 1 and Part 2.

### Colonoscopy

All colonoscopies were performed by 4 experienced gastroenterologists (minimum experience of 1000 procedures) at the Asian Institute of Gastroenterology. A standard protocol for insertion, withdrawal and observation was followed. All colonoscopies were performed using video colonoscopes (CF 145, Olympus, Japan) under moderate propofol sedation. The colon segment including rectum and extending up to the splenic flexure was termed as descending colon; the segment between splenic flexure and hepatic flexure was termed transverse colon; while colon segment proximal to hepatic flexure, including the cecum was termed right colon for the purpose of this study. A complete colonoscopy was defined as reaching the caecum determined by the visualization and documentation of the ileocaecal valve and appendicular orifice.

During the insertion of the scope, two representative pictures were taken from each of these three segments to document the colon prep. All colonoscopies were done the same day of preparation. An early colonoscopy was defined as a procedure initiated before 4 pm while those started after 4 pm were considered late procedure.

### Colon cleansing scale

We used Boston bowel prep scale (BBPS) score to evaluate the adequacy of the preparation [17, 18]. Each colonic segment was graded from 0 to 3 based on quality of the prep. Aggregate score was obtained by adding the score for all 3 segments, thus resulting in a score between 0 and 9. A score ≤ 4 was considered a poor prep, resulting in recommendation for a repeat procedure. However, the patients were evaluated only on the scores for the first procedure and the repeat procedure was not scored for the purpose of the study. A score of 8–9 was considered excellent prep while a score of 5–7 was considered adequate prep. The colon prep was graded by analyzing photo documentation obtained during a colonoscopy by four gastroenterologists (RB, MT, HC, SA and NS) randomized to the study groups.

### Inter-observer agreement

All endoscopists Participating in the study were given a half an hour presentation on Boston bowel prep scores and on photo documentation of prep. A calibration exercise was performed for the gastroenterologists involved in scoring the prep.

### Statistical analysis

In Part 1, colonoscopy prep scores between 2 L PEG without or with Lubiprostone (Group A and B) were evaluated. A sample size of at least 200 subjects was required to give an 80 % power at a 2 sided α of 0.05 to detect a 3 point difference in the BBPS score. In Part 2, the efficacy of bowel prep regimens containing lubiprostone with varying volumes of PEG namely 2, 1.5 and 1 L (Group B, C and D) were evaluated. A Chi-square test was used for nominal data while student’s *t*-test or ANOVA was used for continuous data. For non-parametric data, Man-Whitney *U*-test (for 2 groups) or Kruskal-Wallis test (for 3 groups) were used. A 2 tailed *p* value < 0.05 was considered significant. All study members had access to the study data and reviewed the results.

## Results

### Part I

A total of 600 subjects were screened. 56 patients did not meet the inclusion criteria, 84 declined to Participate. 2 subjects did not come back for procedure.

Initially 458 patients were enrolled in the study of which 228 patients were allocated to group A and 230 patients were allocated to group B. 16 patients (7 from group A and 9 from Group B) in whom colonoscopy could not be completed were excluded from the analysis. The indications for colonoscopy were primarily chronic lower abdominal pain and bloody diarrhoea. There was no significant difference in indications for colonoscopy between the two groups (Table 1) . The average age was 45.8 years, average body weight 62.6 Kg and 71 % patients were male. The baseline characteristics of age, gender, height, weight, comorbid illness including diabetes and dietary habits were similar in both the groups (Table 2).

**Table 1:** Indications for colonoscopy

	PEG + Placebo Group A (*n* = 221)	PEG + LB Group B (*n* = 221)
Constipation	16.6 %(37)	15 %(33)
Chronic lower abdominal pain	31 %(69)	34 %(75)
Diarrhea	8.8 %(19)	10 %(22)
Blood in motions	21 %(46)	17.5 %(39)
Unexplained weight loss	1.9 %(4)	1 %(2)
Anemia under evaluation	2.7 %(6)	2.5 %(6)
IBD follow up	14 %(31)	16 %(36)
Family H/O CRC or polyposis	1.2 %(3)	0.8 %(2)
Evaluation of abnormality by other imaging	2 %(4)	1.8 %(4)
Others	0.8 % (2)	1 %(2)

**Table 2:** Baseline characteristics of subjects

	PEG + Placebo	PEG + Lubiprostone
	Group A (*n* = 221)	Group B (*n* = 221)
Age, Mean (SD)	45.8 (14.7)	45.9 (15.2)
Male, n (%)	154 (69.7 %)	160 (72.4 %)
Height in cm, Mean (SD)	160.9 (9.0)	161.4 (10.1)
Weight, Kg, Mean (SD)	63.3 (13.7)	61.9 (14.3)
College graduate, n (%)	95 (42.9 %)	122 (55.2 %)
Vegetarian Diet, n (%)	31 (27.6 %)	70 (31.6 %)
Rice based diet, n (%)	190 (86 %)	193 (83.7 %)
Prior colonoscopy, n (%)	60 (27.6 %)	52 (23.5 %)
Co-morbid illness, n (%)	94 (42.5 %)	79 (35.7 %)

**Fig. 2:**
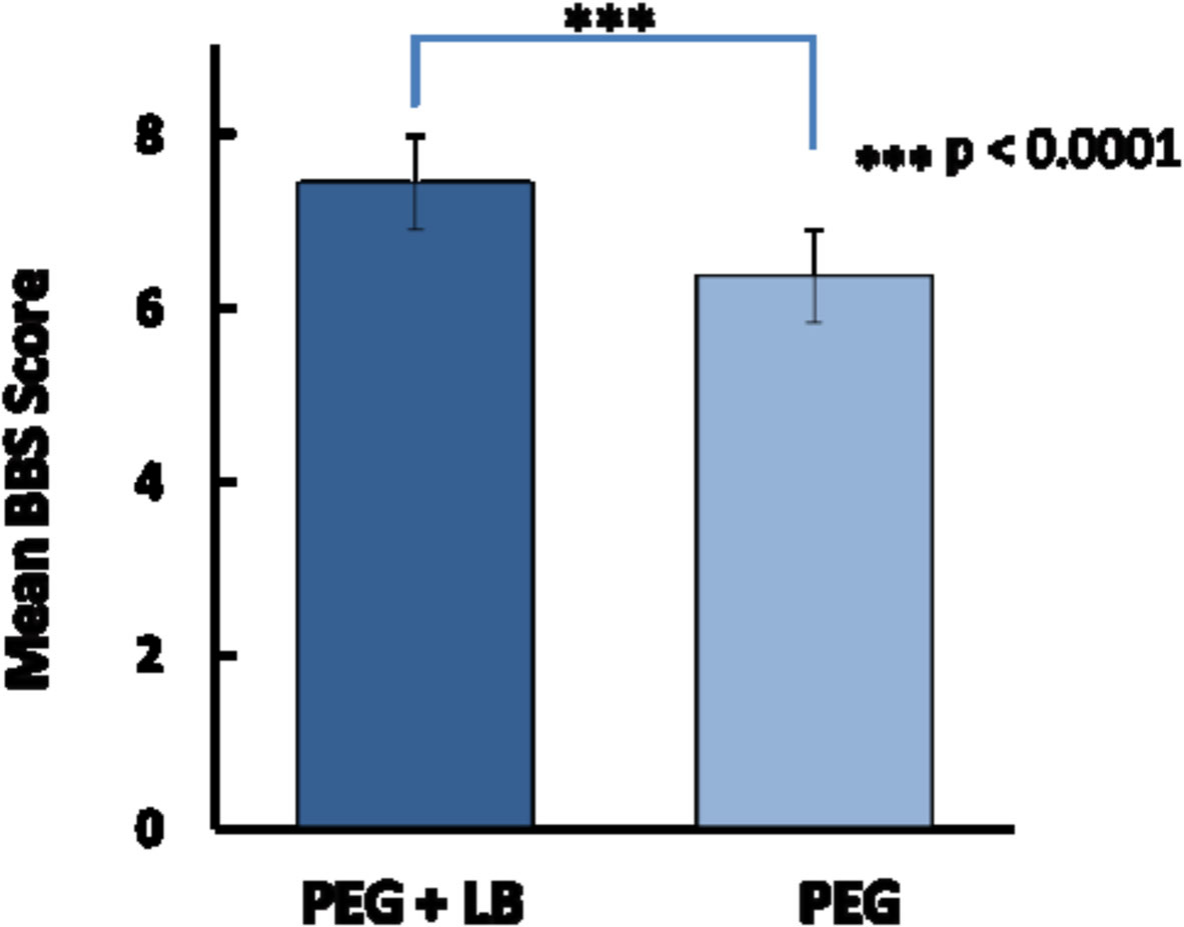
Mean BBPS score with and without Lubiprostone prior to PEG preparation

**Fig. 3:**
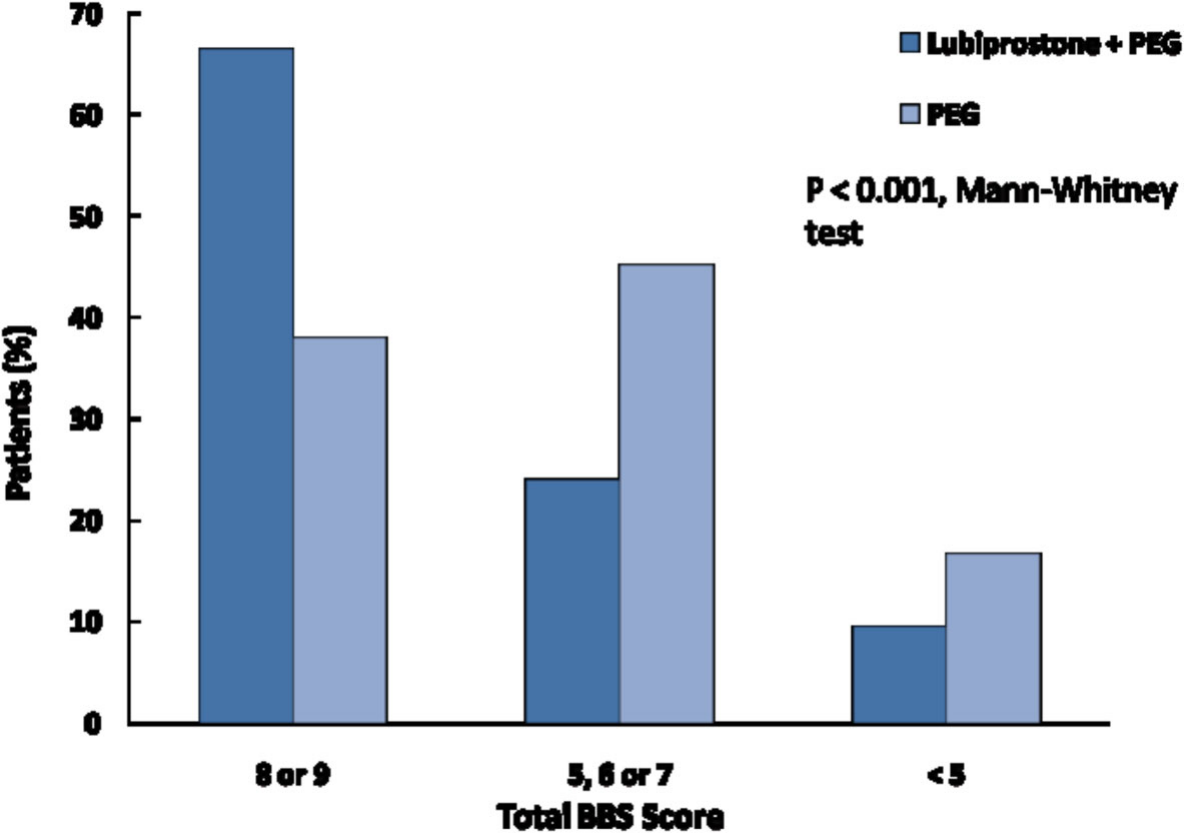
Quality of preparation with the addition of Lubiprostone (BBS score 8-9: excellent; 5–7: adequate; <4: poor prep/repeat)

**Fig. 4:**
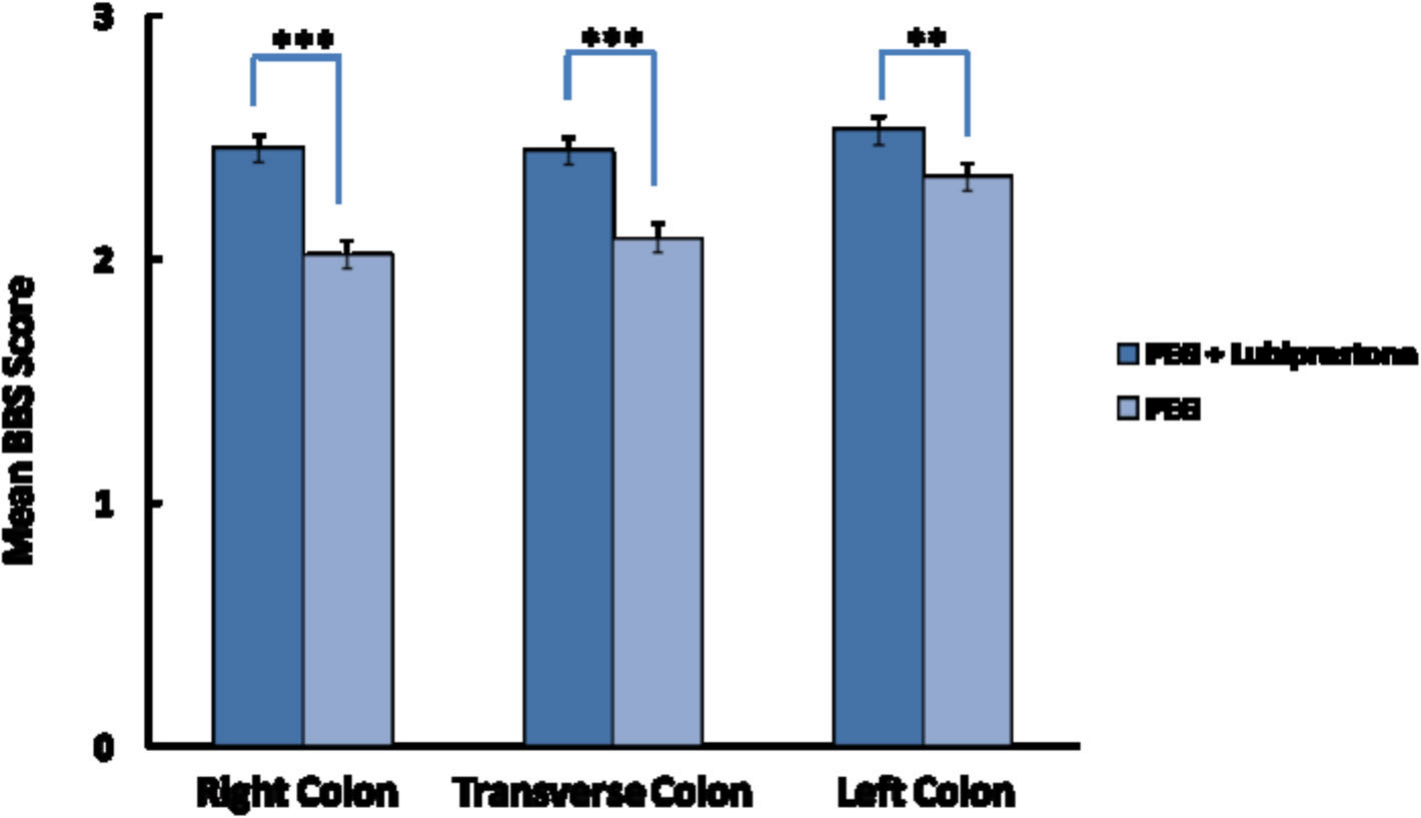
Improved BBPS scores in all three segments of colon including the right colon with addition of Lubiprostone

Addition of lubiprostone resulted in significant improvement in total BBPS score (7.44 ± 0.14 vs. 6.36 ± 0.16, Mean ± SE, *p* < 0.0001) (Fig. 2). 66.5 % patient in group B and 38 % patients in group A had excellent prep, 24 % in group A and 42.5 % in group B had adequate prep while 9.5 % patients in group B and 16.7 % patients in Group A had poor prep (*P* < 0.01) (Fig. 3). Overall 90.5 % patients in group B and 83.3 % patients in group A had excellent or adequate prep (*P* < 0.01). Lubiprostone improved BBP scores in all three segments of colon including the right colon (Fig. 4).

### Part II

Given the significant improvement in bowel prep quality with addition of lubiprostone, we undertook the second Part of the study where we added two arms with reduced doses of PEG; 1.5 L PEG with LB (Group C) and 1 L PEG with LB(Group D). The investigators were blinded to the allocation.

A total of 75 patients enrolled in group C and 71 patients in group D were enrolled in to the second part. As the inclusion, exclusion criteria and methods were similar we also included Group B (lubiprostone with 2 L PEG) in this comparison. At baseline age, gender and dietary habits were similar in all three groups. Group D had less college graduates compared to group B and C. Significantly less number of patients had diabetes and comorbid illness in group B compared to the other two groups.

**Fig. 5:**
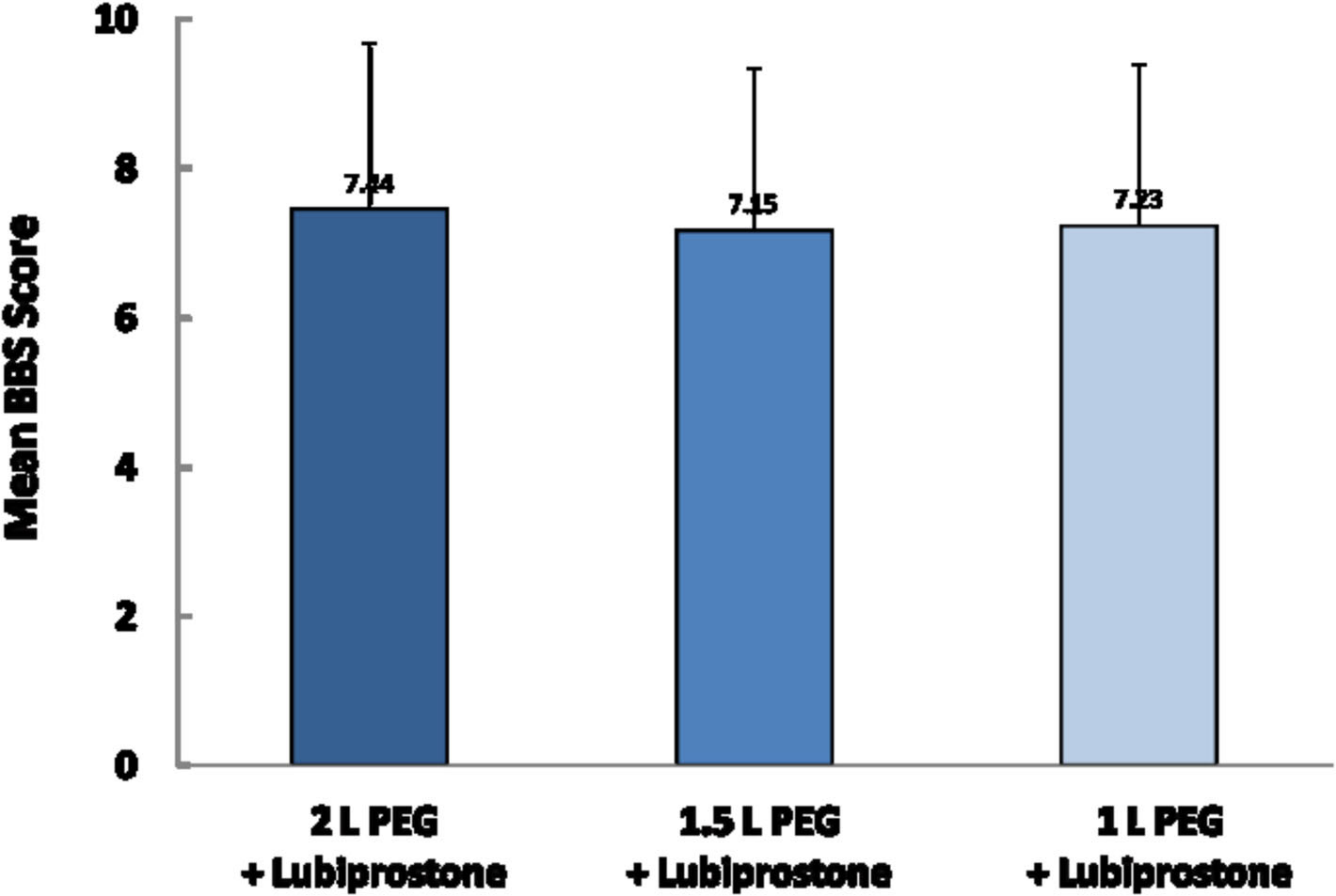
Mean BBPS scores with 2, 1.5 and 1 L volume of PEG on addition of Lubiprostone

**Fig. 6:**
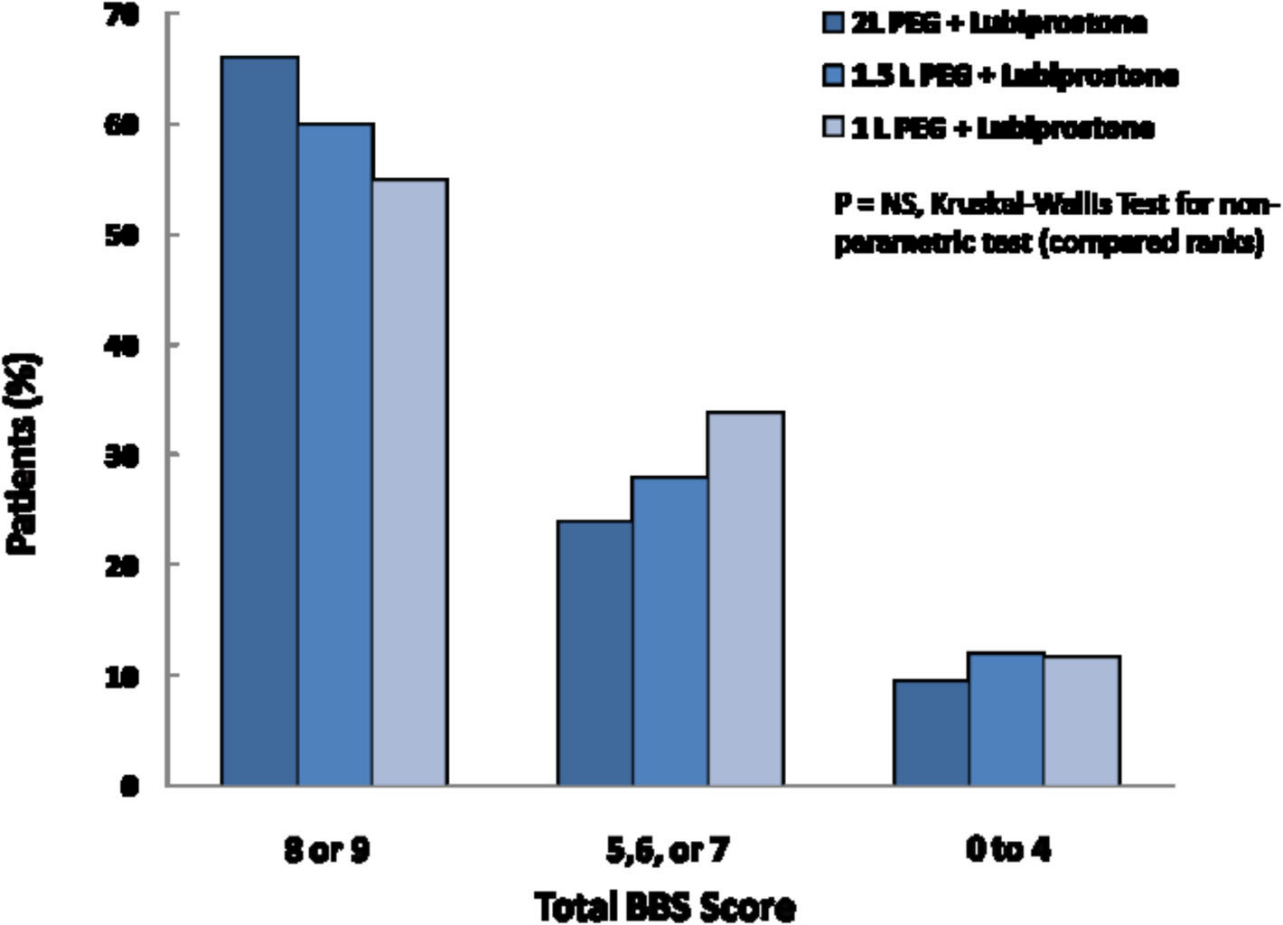
Quality of preparation (BBP score) with 2, 1.5 & 1 L PEG preparations. Note: No significant difference in poor preparations/repeat procedures with lower doses

No difference was seen in the over-all colonoscopy prep scores across the three groups. Mean BBPS scores were 7.44 ± 0.14, 7.30 ± 0.25 and 7.25 ± 0.26 in group B,C and D respectively (*p* > 0.05) (Fig. 5). There was no difference in patients with poor prep requiring repeat procedures across the three groups (9.5, 12, and 11.7 % in group B, C and D respectively, *p* > 0.05) (Fig. 6). There was a non-significant trend towards excellent prep with higher PEG dose (66, 60 and 54.9 % in group B, C and D). The decrease in proportion of patients with excellent prep in lower PEG dose was replaced by higher proportion of patients with adequate prep in those groups (24, 28 and 33.8 % in group B, C and D respectively).

## Discussion

It is widely accepted that the quality of bowel preparation has a direct bearing on the diagnostic accuracy and therapeutic safety of colonoscopy [4, 19, 20]. Recent studies have shown an adenoma miss rate of 33 to 46 % in those with inadequate bowel preparation at the time of their screening colonoscopy [19, 20] and is considered to be one of the major factors associated with post colonoscopy colorectal cancer [8].

This study addresses three important issues relevant to bowel preparation: a) the quality of preparation b) the reduced PEG dosage needed for adequate preparation & c) effective same day preparation. We have assessed the adequacy of LB pretreatment to standard PEG preparation for colonoscopy. The addition of LB produced a significantly better colon cleansing throughout the colon including the right colon. The number of repeat procedures was also significantly lower compared to that of the PEG only group. This demonstrates that LB could be an important adjunct to standard bowel preparation to allow a consistent & reliable colonoscopy.

Previous clinical studies have shown a success rate for bowel cleansing with PEG based preparations ranging from 56 to 76 % [21, 22]. In our study it was 90.3 % with addition of LB. This suggests that LB provided an additional laxative effect resulting in the significantly better preparation. The accelerated small intestinal and colonic transit time with LB with increased bowel movement frequency could have contributed to the efficacy.

The large volume of PEG based formulations with resultant distension, nausea and vomiting is currently the largest barrier for patient acceptance & compliance. Low volume PEG formulations represent an important alternative to standard volume preparations to reduce the discomfort and inconvenience of bowel preparations [23, 24]. Studies have shown comparable results for low volume PEG formulations with respect to ease of consumption, acceptability, or the endoscopists’ satisfaction with the quality of bowel preparation [25–29]. A low volume PEG preparation with ascorbic acid has been shown to be an effective alternative to the standard 4 L PEG [30]. However prokinetics like cisapride did not improve bowel cleansing though the nausea and vomiting was reduced [31]. Results with the addition of Tegaserod, a 5HT receptor agonist was also discouraging with no change in tolerance, effectiveness or adverse effects [32]. A combination therapy with Bisacodyl (same cost as LB) is available but the safety profile has been doubted [33, 34]. In fact the initial combination therapy with 20 mg Bisacodyl has been reduced to 5 mg due to adverse side effects including the risk of ischaemic colitis [34, 35].

Strengel et al. have earlier used LB as a pretreatment for split dose colonoscopy preparation in 200 patients for CRC screening with improved cleansing quality [36]. Split dosing has been recommended to improve patient tolerance [37–41]. However Ell C et al. in a meta-analysis have demonstrated that even single dosing intake was effective in more than 70 % of patients. The timing of the intake could therefore be decided based on the patient’s convenience [42]. Our findings appear similar. Additionally, our study is noteworthy since we included all symptomatic patients referred for diagnostic colonoscopy and was not limited to colorectal cancer screening. No dietary restrictions were advocated. A single time preparation of 2- L alone was given and majority of procedures were done on the same day. Cleanliness in the right colon was less satisfactory with low volume 2 L PEG than with 4 L PEG in a few studies [43, 44]. However in our study there was better cleansing of the right colon as well.

Same-day bowel cleansing for colonoscopy has been practiced in parts of Asia for many years. Endoscopists in the western world are now enthusiastically evaluating this approach evidenced by an increased number of reports describing same-day preparation. A number of studies have shown the benefit of taking the whole preparation in the morning of a scheduled afternoon colonoscopy. Matro R et al. have shown that the overall efficacy was same between morning only and split dosing of bowel preparation [45]. Gurudu SR et al. retrospectively reviewed the colonoscopy records of 1345 subjects who underwent afternoon colonoscopy. They concluded that a same-day PEG bowel preparation yields significantly improved colonic preparation for afternoon colonoscopy compared with preparations completed the evening prior to examination [46]. In fact the US Multi-Society Task Force on Colorectal Cancer has recently recommended same-day regimen as an acceptable alternative to split dosing, especially for patients undergoing an afternoon examination [47].

Our study is the first study to demonstrate that even reduced doses of PEG & same day preparation can produce adequate bowel cleansing with the addition of LB. This use of a more practical single day low volume PEG regimen could definitely improve patient compliance to CRC screening guidelines in the future. In fact, same day preparation has recently been recommended as a useful tip for a better colonoscopy [48, 49].

This study does have some limitations. It is a single center study where only outpatients were recruited. The relatively mobile patients with detailed explanation of the bowel preparation methods prior to the procedure could have improved compliance and the results. It was not an outcome study and as such the lesion miss rate was not evaluated. We are unable to comment whether improved bowel cleansing is associated with an increased adenoma detection rate. However BPSS scores have been shown to correlate with polyp detection rate in earlier studies [17].

Colonoscopy is now the accepted standard for large bowel evaluation. Screening colonoscopies with removal/sampling of adenomatous polyps have reduced the incidence and mortality from colorectal cancer. However the hassles of a large volume bowel preparation, the preprocedure dietary restrictions have significantly impacted patient compliance and tolerability. Our study highlights that pretreatment with a single dose of Lubiprostone can improve bowel cleansing with significantly reduced rate of repeat procedures. Even reduced doses of PEG could be used with no impact on overall quality of preparation. This study is a step forward towards easy same day colonoscopy. Further validation studies in different population cohorts are required before incorporation into standard colon preparation guidelines.

## Conclusion

Single dose LB prior to PEG significantly enhanced bowel preparation compared to PEG alone. There was no significant difference in quality of preparation with lower doses of PEG when combined with LB.

## Abbreviations

BBPS: Boston bowel prep scale
L: Liter
LB: Lubiprostone
NPO: Nil per oral
PEG: Polyethylene glycol
